# Monitoring urinary collagen metabolite changes following collagen peptide ingestion and physical activity using ELISA with anti active collagen oligopeptide antibody

**DOI:** 10.1038/s41598-021-92934-1

**Published:** 2021-06-29

**Authors:** Yoshihiro Osawa, Kaho Nomura, Yoshifumi Kimira, Seiji Kushibe, Ken-ichi Takeyama, Masashi Nagao, Aya Kataoka-Matsushita, Seiko Koizumi, Hiroshi Mano

**Affiliations:** 1 Department of Clinical Dietetics and Human Nutrition, Faculty of Pharmacy and Pharmaceutical Sciences, Josai University, Saitama, Japan; 2 Department of Management, Faculty of Management, Josai University, Saitama, Japan; 3 Research Institute of Agriculture and Life Sciences, Tokyo University of Agriculture, Tokyo, Japan; 4 Graduate School of Health and Sports Science, Juntendo University, Chiba, Japan; 5 Nitta Gelatin Inc, Osaka, Japan

**Keywords:** Biomarkers, Endocrinology

## Abstract

Active collagen oligopeptides (ACOP) are bioactive collagen-derived peptides detected by a recently-established ELISA. To facilitate studies of the function and metabolism of these products, this study aims to determine which of these peptides is recognized by a novel anti-ACOP antibody used in this ELISA. We then investigate the effect of collagen peptide (CP) ingestion and exercise on urinary ACOP concentrations in a cohort of university student athletes using colorimetric, LC–MS/MS, and ELISA. We observed that the antibody showed strong cross-reactivity to Pro-Hyp and Gly-Pro-Hyp and weak cross-reactivity to commercial CP. CP ingestion increased the urinary level of ACOP over time, which correlated highly with urinary levels of peptide forms of Hyp and Pro-Hyp. Physical activity significantly decreased the urinary ACOP level. This study demonstrates changes in urinary ACOP following oral CP intake and physical activity using ELISA with the novel anti-ACOP antibody. Thus, ACOP may be useful as a new biomarker for collagen metabolism.

## Introduction

Collagen, which accounts for approximately 30% of the total protein in animals, is an important component of the extracellular matrix that is essential for cell adhesion and function^1^. Collagen homeostasis in tissues is maintained by collagen turnover^2^. Among the metabolites produced during this process are bioactive collagen peptides (CP) resulting from its enzymatic degradation^3,4^. These collagen peptides are efficiently absorbed in the digestive tract, appearing in the blood within a few hours of the ingestion of collagen-containing substances^5^.

Oral intake of commercially available CP is reported to have physiological effects such as promoting skin pressure ulcer recovery^6^, stimulating bone formation and inhibiting bone resorption^7^, inhibiting arthritis^8^, increasing fat free body mass and grip strength^9^, and preventing atherosclerosis^10^. Recent studies report that prolylhydroxyproline (Pro-Hyp), one of the final collagen metabolites, stimulates the proliferation and differentiation of skin fibroblasts^11^, chondrocytes^12^, and osteocytes^13^. The detailed mechanism underlying the bioactivity of CP is unclear. However, evidence indicates that Pro-Hyp is taken up by mesenchymal cells via peptide transporters^14^ and subsequently plays a role in regulating cell function as a binding factor for nuclear receptors^15^.

During biosynthesis, collagen undergoes post-translational modifications including hydroxylation, glycation, and the formation of intra- and intermolecular crosslinks^16^. Collagen turnover, involving biosynthesis and degradation, is altered by the effects of aging^17^, physical activity^18^, and nutritional status^19^. These factors may also affect the status of collagen metabolites in the body. Therefore, the investigation of CP within the body may be very important to understanding its bioactivity.

Serum and urinary levels of collagen-derived peptides can be used to monitor collagen metabolism. Collagen-derived amino acids and di- and tripeptides are identified and quantified using liquid chromatography^20^, gas chromatography^21^, and mass spectrometry^22^, a highly selective technique known as LC–MS/MS. A recently developed, highly sensitive ELISA also detects bioactive CP. This assay does not require dedicated equipment and can be used to analyze many samples simultaneously in a short time. In this study, we determine the specific active collagen oligopeptides (ACOP) detected by this ELISA for use in monitoring collagen metabolism. We then investigate the effect of CP ingestion and exercise on urinary ACOP concentrations in a cohort of university student athletes using colorimetric, LC–MS/MS, and ELISA.

## Results

### Cross-reactivity of anti-ACOP antibody

**Figure 1:**
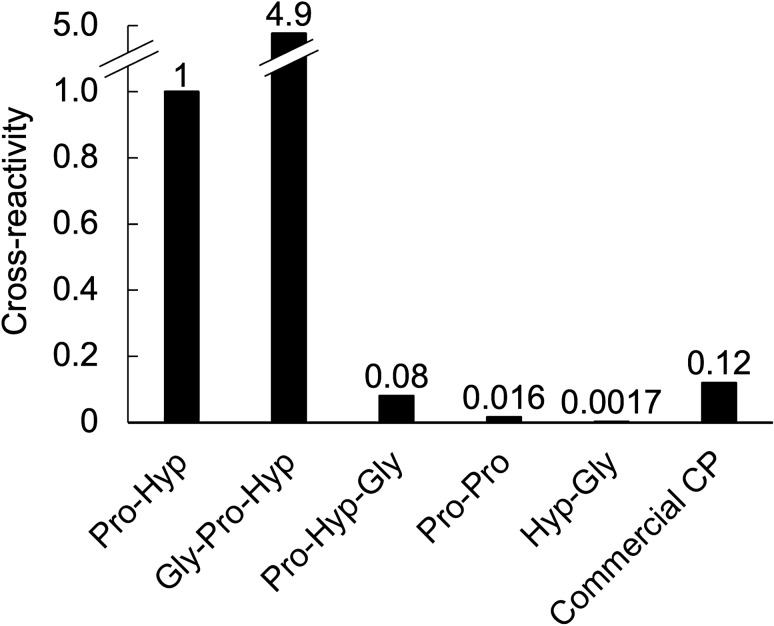
Cross-reactivity of anti-ACOP antibody to five collagen-derived peptides. Data are presented as relative values per weight normalized to that of Pro-Hyp.

ELISA was used to investigate the cross-reactivity of anti-ACOP antibody with six different peptides: Pro-Hyp, Gly-Pro-Hyp, Pro-Hyp-Gly, Pro-Pro, Hyp-Gly, and commercial CP. In this ELISA, the value measured per weight was calculated for each sample relative to that of Pro-Hyp. As shown in Fig. 1, the cross-reactivity was approximately fivefold for Gly-Pro-Hyp and approximately 0.1 to 0.001-fold for Pro-Hyp-Gly, Pro-Pro, and Hyp-Gly. The cross-reactivity of commercial CP, the hydrolysis product of collagen, was approximately 0.1-fold. These results show that the anti-ACOP antibody had particularly strong cross-reactivity with Pro-Hyp and Gly-Pro-Hyp, binding not only to dipeptides and tripeptides but also to the collagen-derived oligopeptide CP.

### Urinary level of ACOP after collagen peptide (CP) ingestion

**Figure 2:**
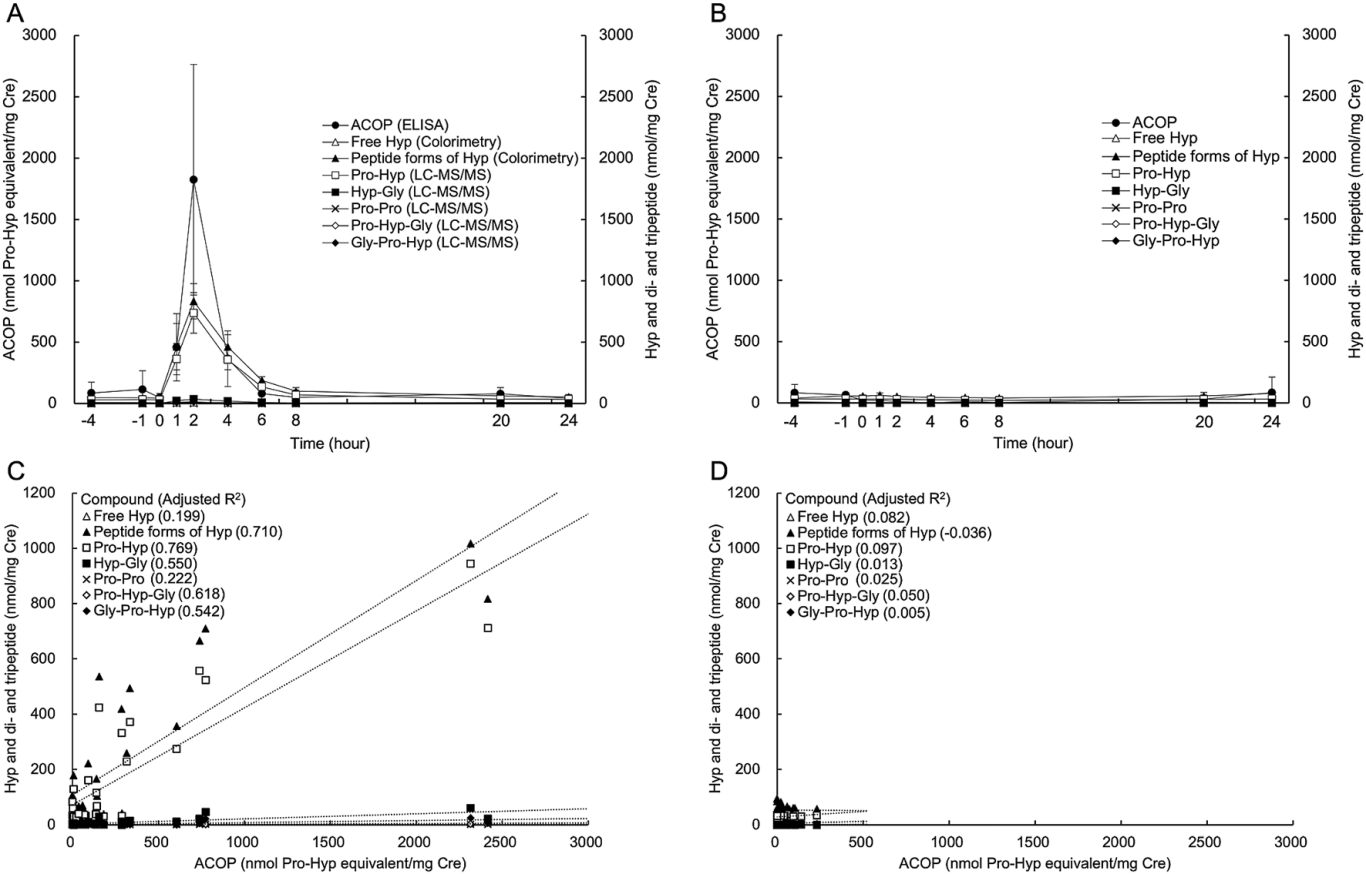
Urinary levels of collagen-derived metabolites after CP ingestion. Time course of collagen-derived metabolite levels in the group of CP ingestion (**A**) and control (**B**). Correlation between ACOP and each collagen-derived metabolite in the group of CP ingestion (**C**) and control (**D**). Samples were measured using colorimetry for free Hyp and peptide forms of Hyp; LC–MS/MS for Pro-Hyp, Hyp-Gly, Pro-Pro, Pro-Hyp-Gly, and Gly-Pro-Hyp; and ELISA for ACOP. Each symbol represents the mean concentration and the bar represents the standard deviation (n = 3, Mean ± standard deviation). The time course consisted of 10 time points over 24 h, with the time of CP ingestion as the 0 h. − 4 and 20 h were defined as the first early morning urine. Dotted lines in **C** and **D** indicate the regression lines for the concentration of each compound, and the adjusted coefficient of determination (adjusted R^2^) at upper left corner. The amount of ACOP was calculated as Pro-Hyp equivalents. Creatinine level was used to correct for variability in concentrations of urinary components.

We observed that the urine levels of seven out of eight collagen degradants (ACOP, Peptide forms of Hyp, Pro-Hyp, Hyp-Gly, Pro-Pro, Pro-Hyp-Gly, and Gly-Pro-Hyp) increased after CP ingestion, with no change in Free Hyp (Fig. 2A). In the group of control, free Hyp in the first early morning urine 4 h before CP intake was only significantly higher, and other collagen degradants were unchanged over time (Fig. 2B). These collagen degradants decreased to approximately 5% or less after 8 h of ingestion compared to each of the peak concentration. Furthermore, as shown in Fig. 2C, an increase in ACOP concentration in the group of CP ingestion strongly correlated with the increase of five collagen degradants except free Hyp and Pro-Pro, especially peptide forms of Hyp and Pro-Hyp. As shown in Fig. 2D, ACOP showed no correlation with any of the collagen degradants in the group of control. These results show that ingested CP is metabolized to form ACOP, which is subsequently excreted in the urine together with Pro-Hyp and peptide forms of Hyp.

### Urinary level of ACOP before and after physical activity

**Figure 3:**
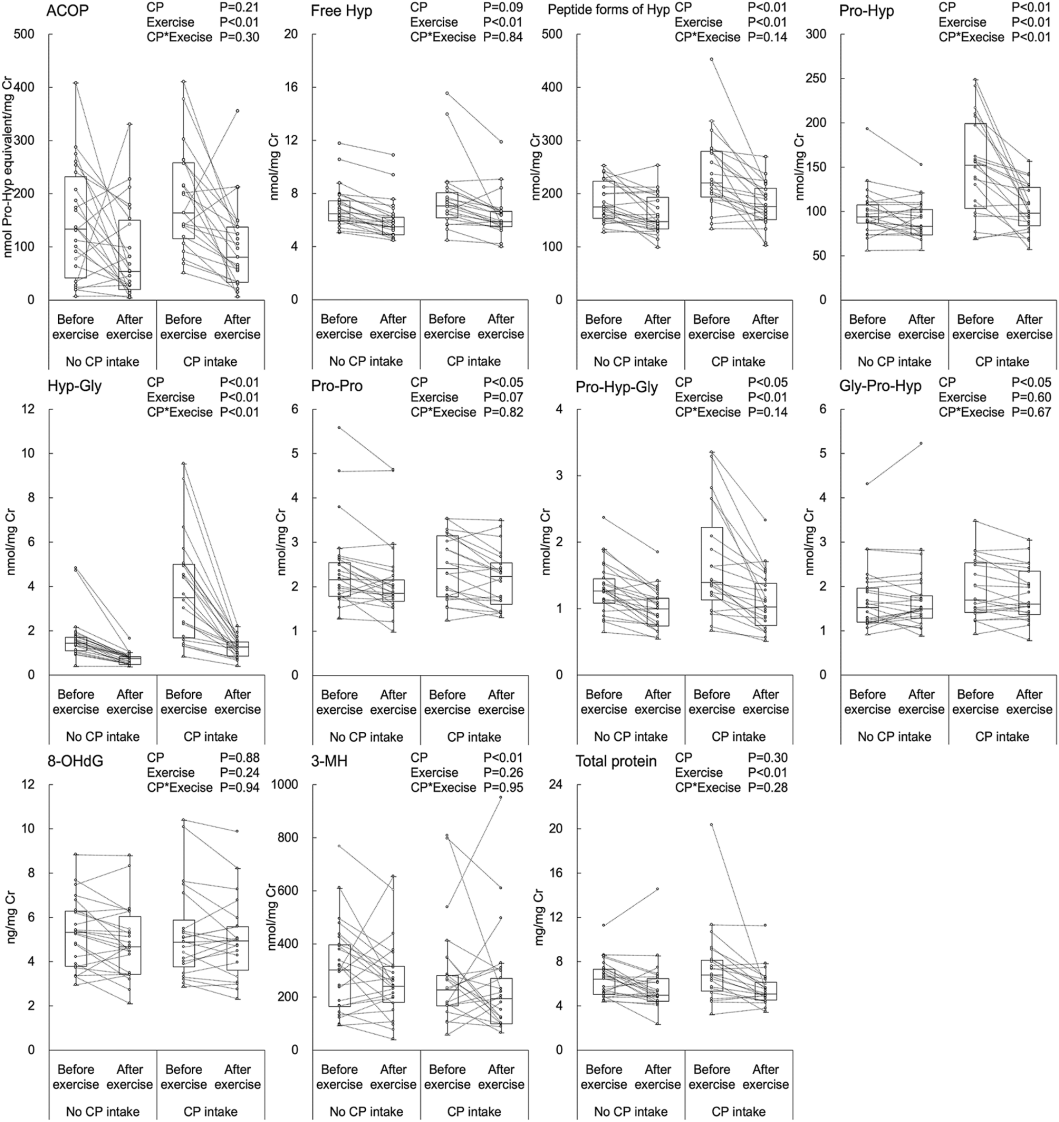
Effect of physical activity on urinary collagen-derived metabolites. Boxplots indicate the median, interquartile range, maximum–minimum whiskers, and outliers. Circles and dotted lines indicate differences between values measured before and after exercise in athletes who did (n = 22, CP intake) and did not (n = 24, No CP intake) routinely take CP supplements. Each sample were measured using colorimetry for free Hyp, peptide forms of Hyp and total protein; LC–MS/MS for Pro-Hyp, Hyp-Gly, Pro-Pro, Pro-Hyp-Gly, Gly-Pro-Hyp, 8-hydroxy-2′-deoxyguanosine (8-OHdG), and 3-methylhistidine (3-MH); and ELISA for ACOP. Two-way analysis of variance (upper right corner of each graph) shows the effect of routine CP intake and physical activity (Exercise). P < 0.05 was considered statistically significant.

Next, we investigated the effect of physical activity on changes in collagen metabolism. Comparison of urinary collagen metabolites both before and after exercise showed a higher level of ACOP than free Hyp, Pro-Pro, Pro-Hyp, Hyp-Gly, Pro-Hyp-Gly, and Gly-Pro-Hyp, but lower than peptide forms of Hyp (Fig. 3). Two-way analysis of variance showed that urine from the group with routine CP intake had significantly higher peptide forms of Hyp, Pro-Hyp, Hyp-Gly, Pro-Pro, Pro-Hyp-Gly, and Gly-Pro-Hyp and significantly lower levels of 3-methylhistidine (3-MH) (Fig. 3). Physical activity decreased urinary ACOP, free Hyp, peptide forms of Hyp, Pro-Hyp, Hyp-Gly, Pro-Hyp-Gly, and total protein, with the greatest difference observed in ACOP. Pro-Hyp and Hyp-Gly showed an interaction effect, and a synergistic effect was observed, which was decreased by CP intake and physical activity. 8-hydroxy-2’-deoxyguanosine (8-OHdG) did not differ CP intake and before and after exercise. These results show that the urinary ACOP level was decreased by physical activity.

## Discussion

The main purpose of this study was to investigate the effects of CP intake and physical activity on ACOP metabolism. We observed that the anti-ACOP antibody crossreacted with five di- and tripeptides and CP derived from collagen, with strong affinity for Pro-Hyp and Gly-Pro-Hyp and weak affinity to CP. Type I collagen, the source of CP, contains approximately forty Pro-Hyp sequences per molecule^1^. Thus, ELISA using this antibody was able to specifically detect ACOP with the Pro-Hyp sequence.

We observed that urinary ACOP increased over time after CP ingestion and correlated highly positively with Pro-Hyp and peptide forms of Hyp levels. Several collagen-derived di- and tripeptides have been reported to increase in blood and urine for several hours after CP ingestion^22^. These collagen-derived metabolites have been used as biomarkers to determine their digestibility and absorption^23,24^. The current results are consistent with these previous studies, demonstrating that this ELISA method can be used to assess changes in urinary ACOP.

In the group of control with a collagen-free diet and no CP ingestion, ACOP was not correlated with any of the collagen degradants. Furthermore, ACOP and collagen-derived di- and tripeptides showed no diurnal variation. Collagen turnover is known to have an endogenous circadian rhythm. The carboxy-terminal propeptide of type I collagen (PICP) and the carboxy-terminal pyridinoline cross-linked telopeptide of type I collagen (ICTP) have been found to be approximately 20% higher at night than during the day^25^. Changes in endogenous collagen derived urinary ACOP are unaffected by these factors and may behave differently from these biomarkers.

Physical activity significantly decreased urinary ACOP levels. This finding is consistent with a previous study reporting a decrease in urinary Hyp level immediately after physical activity^26^. We observed that ACOP was unique among the CP metabolites in that the concentration before and after physical activity differed much more than that of Free Hyp or Pro-Hyp-Gly.

Surprisingly, physical activity did not increase the total urinary protein level. In general, hard physical activity decreases the filtration function of the kidney glomeruli and increases the excretion of proteins in urine^27^. We observed that the levels of 8-OHdG, an oxidative stress marker^28^ and 3-MH, a muscle tissue damage marker^29^, were unchanged, indicating that the exercise load in this study was not taxing for the athletes and did not cause kidney or muscle tissue damage.

Our investigation of the effect of oral CP intake on urinary metabolites showed that the ACOP level was little effect after approximately 12 h from the CP intake. A previous study reports that the amount of peptide forms of Hyp in blood and urine, especially Pro-Hyp, increases for several hours after CP ingestion^22^. Similarly, we found that urinary peptide forms of Hyp and Pro-Hyp levels increased significantly after CP intake. These results show that the response to CP intake differs between ACOP and Pro-Hyp, even though Pro-Hyp is a major component of ACOP.

We found that ACOP differed from the other collagen metabolites in several ways. Urinary ACOP was present in greater amounts, less affected by oral CP intake, and more affected by physical activity. Furthermore, ACOP can be easily measured in a short time using ELISA, which can be used to analyze urine samples without pretreatment such as cumbersome hydrolysis or expensive equipment as in LC–MS/MS. Taken together, ACOP may be a suitable marker for the evaluation of collagen metabolism in terms of novelty and convenience.

Interestingly, the decrease in urinary Pro-Hyp level following physical activity was observed only in the group that routinely took CP. This discovery suggests that active collagen oligopeptides may prevent exercise-induced depletion of Pro-Hyp. The production of Pro-Hyp from ACOP may be a faster and more flexible response to changes in the body’s demand for Pro-Hyp than that from collagen. Experiments in animal models using radioisotopes have reported that orally administered Pro-Hyp is rapidly metabolized and absorbed into specific tissues^14^. Most of these Pro-Hyp–derived metabolites are unidentified, but some are reported to be converted into cyclic Pro-Hyp^30^. Our findings indicate that ACOP is involved in such Pro-Hyp metabolism. Therefore, dietary ACOP supplementation may be useful for rapid compensation for Pro-Hyp consumption during physical exertion.

This study has several limitations. First, the cross-reactivity of the anti-ACOP antibody used in this study was confirmed only for five di- and tripeptides. Therefore, the behavior and strength of its reactivity to other CP and non-CP-derived peptides remains unknown. Second, the present results were obtained in a cohort of athletes. In general, the diet, physical activity, and body composition of athletes differ from those of non-athletes; therefore, the urinary ACOP level observed in this study and its changes in response to CP intake and physical activity cannot be directly applied to non-athletes.

In conclusion, the present study demonstrated that ACOP was less affected by CP intake and clearly decreased by physical activity compared to peptide forms of Hyp and Pro-Hyp. Therefore, urinary ACOP detected by ELISA using anti-ACOP antibody could be a new indicator for monitoring changes in collagen metabolism. However, it is unknown what the changes in the amount of ACOP excreted in the urine means. In addition, the molecular profiles and mechanisms of the changed ACOP remain unresolved in detail. Further research is needed to understand the physiological role of active collagen oligopeptides.

## Methods

### Chemicals

Hydroxyproline (Hyp), 8-hydroxy-2′-deoxyguanosine (8-OHdG), 3-methylhistidine (3-MH) and creatinine were purchased from FUJIFILM Wako Pure Chemical (Osaka, Japan). Pro-Hyp, Hyp-Gly, Pro-Pro, Pro-Hyp-Gly, and Gly-Pro-Hyp were purchased from GL Biochem (Shanghai, China). The fish-derived low-molecular weight commercial collagen peptide (CP, Wellnex TYPE-S) was purchased from Nitta Gelatin (Osaka, Japan). The Pierce BCA Protein Assay Kit was purchased from Bio-Rad (Hercules, CA, USA). The ELISA kit was purchased from Air Plants Bio (Tokyo, Japan). The other analytical grade chemicals were purchased from FUJIFILM Wako Pure Chemical (Osaka, Japan).

### Study approval

This study was approved by the Ethics Committee of Juntendo University (No. 2018133) and conducted in accordance with the Ethical Guidelines for Medical and Health Research Involving Human Subjects published by the Ministry of Health, Labour and Welfare of Japan and the Declaration of Helsinki. Written informed consent was obtained from all participants.

### Cross-reactivity confirmation

Five collagen-derived peptides (Pro-Hyp, Hyp-Gly, Pro-Pro, Pro-Hyp-Gly, and Gly-Pro-Hyp) and CP were mixed with purified water to a concentration of 20 mg/mL. These samples were analyzed by ELISA for cross-reactivity with the anti-ACOP antibody.

### Urine ACOP concentration before and after CP ingestion

Three healthy, non-athlete male subjects (mean age 36.5 ± 13.2 years, mean body weight 66.9 ± 10.8 kg, mean BMI 23.7 ± 3.3 kg/m^2^) were limited to a low-protein diet for the duration of the experiment and a 12-h fast before the start of the experiment. During this time, the subjects were restricted from taking supplements and medications, and liquid intake was limited to water only. The subjects were then orally administered 10 g of commercial CP, or none dissolved in 200 mL of hot water at approximately 50 °C. The two experiments were conducted with an interval of one day. Urine samples were collected at 10 time points before (− 4, − 1, 0 h) and after (1, 2, 4, 6, 8, 20, 24 h) ingestion, with the urines at 4 h before and 20 h after ingestion being the first early morning urine. Urine samples were stored at − 80 °C, and analyzed for the content of ACOP, collagen-derived di- and tripeptides, Hyp, and creatinine. The concentration of all analyzed urine constituents was normalized to that of creatinine.

### Urine collection before and after physical activity

Forty-six healthy male university students in their teens and twenties were recruited from a running club at Josai University. All subjects received no intervention regarding diet, dietary supplements, medications, hydration intake, or exercise regimen during the study. Results of a dietary questionnaire confirmed that all subjects had eaten the same dormitory food the day before the study. The subjects were divided into two groups according to whether they did (mean age 19.5 ± 1.3, mean body weight 57.6 ± 3.7, mean BMI 19.5 ± 1.3 kg/m^2^, n = 22) or did not (mean age 18.9 ± 1.1, mean body weight 55.9 ± 5.4, mean BMI 19.8 ± 1.1 kg/m^2^, n = 24) take CP routinely. The athletes were provided with a CP supplement, the porcine-derived commercial collagen peptide (Wellnex SCP-5200) from Nitta Gelatin (Osaka, Japan). While the subjects were not asked about their specific CP intake, we did confirm that they had not taken any food and CP-containing supplements after 20:00 the day before. Physical exercise among the cohort consisted of running for a mean duration of 1 h and a distance of approximately 13 km. Urine samples were collected from the subjects before and after physical activity, with the urines before physical activity being the first early morning urine. Urine samples were stored at − 80 °C, and analyzed for the content of ACOP, collagen-derived di- and tripeptides, Hyp, 8-OHdG, 3-MH, total protein, and creatinine. The concentration of all analyzed urine constituents was normalized to that of creatinine. Concentrations before and after physical activity were compared between the two groups.

### ELISA

Active collagen oligopeptide concentration was determined using the ELISA kit according to the manufacturer’s instructions. The concentrations were calculated in terms of Pro-Hyp equivalents using a Pro-Hyp standard.

### LC–MS/MS analysis

LC–MS/MS was used to measure Pro-Hyp, Hyp-Gly, Pro-Pro, Pro-Hyp-Gly, Gly-Pro-Hyp, 8-OHdG, 3-MH, creatinine concentrations. To quantify 3-MH, an aliquot of urine diluted 500-fold in 50 mM ammonium bicarbonate solution was added to a threefold volume of ethanol. Samples were centrifuged at 3000 rpm for 5 min followed by filtration through a 0.22 µm filter. An aliquot of each sample was used for mass spectrometry. Chromatography analysis was performed using an ACQUITY UPLC H-Class Bio system (Waters, Milford, MA, USA) equipped with a Hypersil GOLD PFP column (2.1 × 150 mm, 5 µm; Thermo Fisher Scientific, Waltham, MA, USA). The isocratic elution was performed with 2% methanol/0.2% formic acid/2 mM ammonium acetate in water under the following conditions: sample injection volume, 0.5 µL; flow rate, 0.4 mL/min; elution time, 3.5 min; column temperature, 40 °C. Mass spectrometry was performed using a Xevo TQ-XS quadrupole mass spectrometer (Waters, Milford, MA, USA) under the following conditions: capillary voltage, 1000 V (positive ionization mode); desorption temperature, 500 °C; source temperature, 150 °C. Data were acquired using selective reaction monitoring with m/z transition of 170 > 124 for 3-MH. Data were acquired and quantified using Mass Lynx software (version 4.2, Waters).

To quantify 8-OHdG, each urine sample was diluted tenfold in pure water, centrifuged at 12,000 rpm (10,000×*g*) for 5 min, and filtered using a 0.22 µm filter. An aliquot of each filtrate was analyzed by mass spectrometry. Chromatography analysis was performed with an UPLC proteomics Series (Shimadzu, Kyoto, Japan) equipped with an Inertsustain AQ-C18 (2.1 × 150 mm, 3 µm; GL Sciences, Tokyo, Japan). The. The isocratic elution was performed with 8% acetonitrile/0.1% acetic acid in water under the following conditions: sample injection volume, 10 µL; flow rate, 0.3 mL/min; elution time, 3 min; column temperature, 40 °C. Mass spectrometry was performed using a 4000 QTRAP quadrupole mass spectrometer (AB Sciex, Tokyo, Japan) under the following conditions: ion spray voltage, 5500 V (positive ionization mode); source temperature, 500 °C. Data were acquired using selective reaction monitoring with m/z transition of 284 > 168 for 8-OHdG. Data were acquired and quantified using Analyst software (version 1.5, AB Sciex).

To quantify Pro-Hyp, Hyp-Gly, Pro-Pro, Pro-Hyp-Gly, Gly-Pro-Hyp, and creatinine, an aliquot of each urine sample was added to a fourfold volume of acetonitrile. An aliquot of supernatant of the centrifuged samples at 12,000 rpm (10,000×*g*) for 5 min was dried by centrifugal evaporator, diluted 100-fold in pure water, and filtered using a 0.22 µm filter. An aliquot of each filtrate was analyzed by mass spectrometry. Chromatography analysis was performed with an UPLC proteomics Series (Shimadzu, Kyoto, Japan) equipped with an Inertsustain AQ-C18 (2.1 × 150 mm, 1.9 µm; GL Sciences, Tokyo, Japan). The gradient elution was performed with solution A: 0.1% formic acid/water and solution B: 0.1% formic acid/acetonitorile under the following conditions: sample injection volume, 10 µL; flow rate, 0.15 mL/min; elution time, 0 min: B1%, 5 min: B80%; column temperature, 40 °C. Mass spectrometry was performed using a 4000 QTRAP quadrupole mass spectrometer (AB Sciex, Tokyo, Japan) under the following conditions: ion spray voltage, 5500 V (positive ionization mode); source temperature, 700 °C. Data were acquired using selective reaction monitoring with m/z transition of 229 > 132 for Pro-Hyp, 189 > 68 for Hyp-Gly, 213 > 70 for Pro-Pro, 286 > 189 for Pro-Hyp-Gly, 286 > 155 for Gly-Pro-Hyp, and 114 > 72 for creatinine. Data were acquired and quantified using Analyst software (version 1.7, AB Sciex).

### Colorimetry

Hyp concentrations were determined by colorimetric analysis using the Ehrlich reagent after oxidation with chloramine T^31^. An aliquot of the urine sample was hydrolyzed by treatment with 6 N hydrochloric acid at 110 °C for 24 h and then neutralized sodium hydroxide. The hydrolyzed sample analyzed for Hyp content. Free and total Hyp were determined before and after hydrolysis. The peptide form of Hyp was estimated by subtracting the concentration of free Hyp from that of total Hyp. Total protein concentration was determined using the colorimetric assay kit according to the manufacturer’s instructions.

### Statistical analysis

Statistical analysis was performed using EZR (version 1.54, Saitama Medical Center, JICHI Medical University, Saitama, Japan)^32^. The coefficient of determinations was calculated to determine whether ACOP concentration correlated with other that of collagen metabolites. Two-way analysis of variance was used to examine the effect of routine CP intake and physical activity on urinary components. The Smirnov-Grubbs test was used to detect outliers. P < 0.05 was considered statistically significant.

## References

[1] Ricard-Blum S (2011). The collagen family. Cold Spring Harb. Perspect. Biol..

[2] Sprangers S, Everts V (2019). Molecular pathways of cell-mediated degradation of fibrillar collagen. Matrix Biol..

[3] Husek P, Svagera Z, Vsiansky F, Franekova J, Simek P (2008). Prolyl-hydroxyproline dipeptide in non-hydrolyzed morning urine and its value in postmenopausal osteoporosis. Clin. Chem. Lab. Med..

[4] Kusubata M, Koyama Y, Tometsuka C, Shigemura Y, Sato K (2015). Detection of endogenous and food-derived collagen dipeptide prolylhydroxyproline (Pro-Hyp) in allergic contact dermatitis-affected mouse ear. Biosci. Biotechnol. Biochem..

[5] Iwai K (2005). Identification of food-derived collagen peptides in human blood after oral ingestion of gelatin hydrolysates. J. Agric. Food Chem..

[6] Sugihara F, Inoue N, Venkateswarathirukumara S (2018). Ingestion of bioactive collagen hydrolysates enhanced pressure ulcer healing in a randomized double-blind placebo-controlled clinical study. Sci. Rep..

[7] Koning D, Oesser S, Scharla S, Zdzieblik D, Gollhofer A (2018). Specific collagen peptides improve bone mineral density and bone markers in postmenopausal women: A randomized controlled study. Nutrients.

[8] Kumar S, Sugihara F, Suzuki K, Inoue N, Venkateswarathirukumara S (2015). A double-blind, placebo-controlled, randomised, clinical study on the effectiveness of collagen peptide on osteoarthritis. J. Sci. Food Agric..

[9] Jendricke P, Centner C, Zdzieblik D, Gollhofer A, Koning D (2019). Specific collagen peptides in combination with resistance training improve body composition and regional muscle strength in premenopausal women: A randomized controlled trial. Nutrients.

[10] Igase M (2018). A double-blind, placebo-controlled, randomised clinical study of the effect of pork collagen peptide supplementation on atherosclerosis in healthy older individuals. Biosci. Biotechnol. Biochem..

[11] Ohara H (2010). Collagen-derived dipeptide, proline-hydroxyproline, stimulates cell proliferation and hyaluronic acid synthesis in cultured human dermal fibroblasts. J. Dermatol..

[12] Nakatani S, Mano H, Sampei C, Shimizu J, Wada M (2009). Chondroprotective effect of the bioactive peptide prolyl-hydroxyproline in mouse articular cartilage in vitro and in vivo. Osteoarthr. Cartil..

[13] Kimira Y (2014). Collagen-derived dipeptide prolyl-hydroxyproline promotes differentiation of MC3T3-E1 osteoblastic cells. Biochem. Biophys. Res. Commun..

[14] Kawaguchi T, Nanbu PN, Kurokawa M (2012). Distribution of prolylhydroxyproline and its metabolites after oral administration in rats. Biol. Pharm. Bull..

[15] Nomura K, Kimira Y, Osawa Y, Shimizu J, Kataoka-Matsushita A, Mano H (2019). Collagen-derived dipeptide prolyl hydroxyproline directly binds to foxg1 to change its conformation and inhibit the interaction with runx2. Biosci. Biotechnol. Biochem..

[16] Hennet T (2019). Collagen glycosylation. Curr. Opin. Struct. Biol..

[17] Jerban S (2019). Age-related decrease in collagen proton fraction in tibial tendons estimated by magnetization transfer modeling of ultrashort echo time magnetic resonance imaging (UTE-MRI). Sci. Rep..

[18] Kjaer M (2005). Metabolic activity and collagen turnover in human tendon in response to physical activity. J. Musculoskelet. Neuronal. Interact..

[19] Shaw G, Lee-Barthel A, Ross M, Wang B, Baar K (2017). Vitamin C-enriched gelatin supplementation before intermittent activity augments collagen synthesis. Am. J. Clin. Nutr..

[20] Sim HJ, Moon E, Kim SY, Hong SP (2013). Determination of proline-hydroxyproline dipeptide in rat urine by high-performance anion-exchange chromatography coupled with pulsed amperometric detection. J. Chromatogr. B Anal. Technol. Biomed. Life Sci..

[21] Husek P, Pohlidal A, Slabik D (2002). Rapid screening of urinary proline-hydroxyproline dipeptide in bone turnover studies. J. Chromatogr. B..

[22] Taga Y, Iwasaki Y, Shigemura Y, Mizuno K (2019). Improved in vivo tracking of orally administered collagen hydrolysate using stable isotope labeling and lc-ms techniques. J. Agric. Food Chem..

[23] Yamamoto S, Deguchi K, Onuma M, Numata N, Sakai Y (2016). Absorption and urinary excretion of peptides after collagen tripeptide ingestion in humans. Biol. Pharm. Bull..

[24] Shigemura Y, Kubomura D, Sato Y, Sato K (2014). Dose-dependent changes in the levels of free and peptide forms of hydroxyproline in human plasma after collagen hydrolysate ingestion. Food Chem..

[25] Hassager C, Risteli J, Risteli L, Jensen SB, Christiansen C (1992). Diurnal variation in serum markers of type I collagen synthesis and degradation in healthy premenopausal women. J. Bone Miner. Res..

[26] Malm HT, Ronni-Sivula HM, Viinikka LU, Ylikorkala OR (1993). Marathon ruuning accompanied by transient decreases in urinary calcium and serum osteocalcin levels. Calcif. Tissue Int..

[27] Calles-Escandon J (1984). Influence of exercise on urea, creatinine, and 3-methylhistidine excretion in normal human subjects. Am. J. Physiol..

[28] Wu D (2017). Detection of 8-hydroxydeoxyguanosine (8-ohdg) as a biomarker of oxidative damage in peripheral leukocyte dna by uhplc-ms/ms. J. Chromatogr. B Anal. Technol. Biomed. Life Sci..

[29] Millward DJ, Smith K (2019). The application of stable-isotope tracers to study human musculoskeletal protein turnover: A tale of bag filling and bag enlargement. J. Physiol..

[30] Shigemura Y (2018). A pilot study for the detection of cyclic prolyl-hydroxyproline (pro-hyp) in human blood after ingestion of collagen hydrolysate. Nutrients.

[31] Firschein HE, Shill JP (1966). The determination of total hydroxyproline in urine and bone extracts. Anal. Biochem..

[32] Kanda Y (2013). Investigation of the freely available easy-to-use software ‘EZR’ for medical statistics. Bone Marrow Transplant..

